# Association of elevated serumfree light chains with chronic lymphocytic leukemia and monoclonal B-cell lymphocytosis

**DOI:** 10.1038/s41408-019-0220-x

**Published:** 2019-08-05

**Authors:** Alyssa I. Clay-Gilmour, Abdul R. Rishi, Lynn R. Goldin, Alexandra J. Greenberg-Worisek, Sara J. Achenbach, Kari G. Rabe, Matthew J. Maurer, Neil E. Kay, Tait D. Shanafelt, Timothy G. Call, J. Brice Weinberg, Nicola J. Camp, James R. Cerhan, Jose Leis, Aaron Norman, David L. Murray, S. Vincent Rajkumar, Neil E. Caporaso, Ola Landgren, Mary L. McMaster, Susan L. Slager, Celine M. Vachon

**Affiliations:** 1 Division of Epidemiology, Department of Health Sciences, Mayo Clinic, Rochester, MN USA; 2 Department of Internal Medicine, Mercy Hospital, St. Louis, MO USA; 3 Division of Cancer Epidemiology and Genetics, National Cancer Institute (NCI), National Institutes of Health (NIH), Bethesda, MD USA; 4 Division of Biomedical Statistics and Informatics, Department of Health Sciences Research, Mayo Clinic, Rochester, MN 55905 USA; 5 Division of Hematology, Mayo Clinic, Rochester, MN 55905 USA; 6 Stanford University Medical Center, Department of Medicine/Hematology, Stanford, CA USA; 7 Duke University and V.A. Medical Centers, Durham, NC USA; 8 Department of Medicine, University of Utah and Huntsman Cancer Institute, Salt Lake City, UT USA; 9 Division of Medical Oncology, Mayo Clinic, Phoenix, AZ USA; 10 Laboratory Medicine and Pathology, College of Medicine, Mayo Clinic, Rochester, MN 55905 USA; 11 Myeloma Service, Division of Hematologic Oncology, Memorial Sloan-Kettering Cancer Center, New York, NY USA

**Keywords:** Cancer epidemiology, Epidemiology, Risk factors

## Abstract

Chronic lymphocytic leukemia (CLL) and its precursor, monoclonal B-cell lymphocytosis (MBL), are heritable. Serumfree light-chain (sFLC) measures are a prognostic factor for CLL, but their role in susceptibility to CLL is not clear. We investigated differences between sFLC measurements in pre-treatment serum from five groups to inform the association of sFLC with familial and sporadic CLL: (1) familial CLL (*n* = 154), (2) sporadic CLL (*n* = 302), (3) familial MBL (*n* = 87), (4) unaffected first-degree relatives from CLL/MBL families (*n* = 263), and (5) reference population (*n* = 15,396). The percent of individuals having elevated monoclonal and polyclonal sFLCs was compared using age-stratified and age- and sex-adjusted logistic regression models. In age groups >50 years, monoclonal sFLC elevations were increased in sporadic and familial CLL cases compared to the reference population (*p*’s < 0.05). However, there were no statistically significant differences in sFLC monoclonal or polyclonal elevations between familial and sporadic CLL cases (*p*’s > 0.05). Unaffected relatives and MBL cases from CLL/MBL families, ages >60 years, showed elevated monoclonal sFLC, compared to the reference population (*p*’s < 0.05). This is the first study to demonstrate monoclonal sFLC elevations in CLL cases compared to controls. Monoclonal sFLC levels may provide additional risk information in relatives of CLL probands.

## Introduction

Chronic lymphocytic leukemia (CLL) has an underlying heritable predisposition, with ~10% of individuals affected with CLL reporting a first-degree relative with CLL or a related lymphoproliferative disorder^1–3^. Monoclonal B-cell lymphocytosis (MBL), precursor disease to CLL, is an asymptomatic hematologic condition characterized by small absolute levels of blood B-cell clone and no other signs of a lymphoproliferative disorder^3–7^. In the general population, MBL increases with age with a prevalence of 5–9% in individuals over age 60 years^7^. We have reported MBL to be higher among first-degree relatives from CLL families, occurring at a higher rate in high-risk CLL families, implying a shared inherited risk^4^.

Paraproteins, usually aligned with plasma cell disorders, have also been found to be prevalent in other B-cell malignancies, such as CLL^8^. In 2007, Martin et al.^9^ evaluated the frequency of monoclonal serum-free light-chain (sFLC) measurements in patients with other B-cell malignancies and established abnormal sFLCs can be detected in a substantial proportion of patients with Non-Hodgkin’s lymphoma (NHL) and CLL, and may be a useful clinical tool in the early diagnosis of a B-cell malignancy. Since then other studies have shown that sFLCs and the associated *κ*/*λ* ratio (rFLC) can also be used to inform prognosis, survival, and clinical outcomes in patients with CLL^10–17^. Maurer et al.^13^ found that both monoclonal and polyclonal sFLC elevations were associated with poor overall survival in CLL compared to patients with normal sFLCs, even after adjusting for Rai stage. A prospective study showed an abnormal rFLC can be detected several years before the actual diagnosis of CLL in a significant percentage of patients^18^. Thus, sFLC measurements have been implicated in detection and prognosis of CLL, however, few studies evaluating sFLCs and risk of CLL (or MBL) have been performed.

Further, there is limited evidence on the differences between sporadic and familial CLL, with most studies focusing on prognostic and clinical factors. Stage at initial diagnosis, need for treatment, and survival over a 10-year period have been reported to be similar in sporadic and familial cases^19^. Similarly, no differences in the expression of ZAP-70, CD38, and CD23, and levels of serum β2-microglobulin have been reported^20^. Studies that examined the mutational status of genes in the immunoglobulin heavy chain variable region (IgVH), known to be associated with a better prognosis, found higher frequency of mutated CLL in familial compared to sporadic CLL^21^. Another study found differences in frequency of VH gene segments (4–34, 5–51, 1–69, 4–29, 3–23) in familial vs. sporadic disease^22^. Among familial cases, VH gene segment utilization proved non-random and diverged from the frequencies previously reported among unrelated patients with CLL^22^. However, the association of sFLC with familial and sporadic CLL has not been performed.

In this study, for the first time, we investigated the association of sFLC levels with risk of CLL (or MBL) using CLL cases (familial and sporadic), unaffected relatives, and a large comparable reference population. We hypothesized that pre-treatment sFLC elevation would be higher among familial vs. sporadic CLL, and in CLL cases compared to the reference population. Second, given the known familial nature of CLL, we hypothesized that pre-treatment sFLC would be elevated in the family members of patients with CLL (both unaffected relatives and those with MBL) compared to the reference population.

## Methods

### Study participants

All participants provided written informed consent. The protocol was approved by the Institutional Review Board at each participating center. Individuals with pre-treatment serum samples were eligible. Those known to have died within 1 year of sample date were excluded due to likelihood of underlying disease, which could falsely inflate sFLC levels. Five participant groups (primarily Caucasian) were defined for analyses consisting of three case groups and two control groups: (1) Sporadic CLL cases, (2) Familial CLL cases, (3) Familial MBL cases from CLL / MBL families, (4) Unaffected relatives from CLL/MBL families; and (5) a reference population (Olmsted County controls)^23–26^.

#### Sporadic CLL cases

Sporadic CLL cases consisted of incident CLL cases diagnosed from 2002–2008 without a self-reported family history of CLL and/or MBL and with a pre-treatment sFLC measure in the University of Iowa/Mayo Clinic SPORE Molecular Epidemiology Resource (MER)^13^.

#### Familial CLL cases

Familial CLL cases were selected from families with at least two reported CLL and/or MBL cases. Families were recruited through the Genetic Epidemiology of CLL (GEC) consortium^27^ at six institutions (Mayo Clinic, M.D. Anderson Cancer Center, National Cancer Institute (NCI), University of Minnesota/Minneapolis Veteran Affairs (VA) Medical Center, Duke University/Durham VA Medical Center, and University of Utah). Familial CLL cases diagnosed from 1974–2011 from the GEC consortium with sFLC assessed on pre-treatment blood samples were eligible. Cases with unknown treatment status were excluded.

#### Familial MBL cases

In our GEC families described above, relatives were screened for MBL in accordance with our previous work^4^. MBL status was determined by flow cytometry and was defined at the time of screening. Any blood relative found to have MBL in a family with two or more CLL and/or MBL cases was eligible for inclusion. We did not require a specific degree of relation between cases.

#### Unaffected relatives from CLL/MBL families

Family members with no known CLL, MBL, or any other lymphoproliferative disorder who were a first-degree relative to at least one CLL or MBL case from a familial CLL family were considered unaffected relatives. Primarily, MBL screening was performed on a baseline sample, which was also used for the sFLC measurements; however, there were some samples that did not get screened and were assumed to be unaffected.

#### Reference population: Olmsted County (controls)

The reference population was a previously characterized population-based cohort of Olmsted County residents age 50 years or older^23^. From 1 January, 1995, to 21 November, 2003, serum samples were obtained from 21,462 (76.6%) of 28,038 enumerated Olmsted County residents and tested for monoclonal protein levels and FLC. We refer to this group as “controls” as they have no known diagnosed CLL or precursor disease, MBL.

Of the 21,462 controls, we excluded 577 individuals with known plasma cell disorders, including those with monoclonal gammopathy of undetermined significance (MGUS), because these are the disorders in which clonal elevations of FLC are known to occur^28^. However, we did not exclude light chain (LC)-MGUS, since FLC is used in the definition of this condition. We also excluded 4,096 individuals who declined authorization to participate in research^29^ and 758 who did not have sufficient sample volume to perform the FLC assay. Of the remaining 16,031 subjects, we also excluded individuals with a known lymphoproliferative disorder or precursor (*n* = 62) or a first-degree relative with this condition (*n* = 32) from clinical or research databases. After excluding individuals whose death date was within 1 year of sample date (prevalent disease: *n* = 541), the final control group consisted of 15,396 subjects.

#### Free lightchain testing

Heparinized plasma or serum samples were tested using the sFLC assay (FREELITE™, The Binding Site Ltd., Birmingham, UK) to measure *κ* and *λ* immunoglobulin FLC levels at either the Mayo Clinic or National Cancer Institute (NCI)^23^. Prior studies have shown the high intraclass correlation between FLC measures using fresh and stored serum and for the FREELITE™ kappa and lambda free light chains assay; both EDTA-plasma and lithium heparin-plasma can serve as acceptable substitutes for serum^30,31^. As previously described, the analytic sensitivity for the nephelometric FLC assay is 0.1 mg/L for a monoclonal *κ* or *λ* FLC^32^. For samples analyzed at both NCI and Mayo Clinic, elevated FLC was defined as a *κ* or *λ* level above the reference range (*κ* > 19.4 mg/L, *λ* > 26.3 mg/L)^13,33^. Likewise, an elevated FLC was considered monoclonal if the rFLC (*κ*/*λ*) was outside of the normal range of 0.26–1.65^13,33^. Elevated FLC levels with normal rFLC (*κ*/*λ*) were considered as polyclonal. Patients with normal FLC levels but an abnormal rFLC (*κ*/*λ*) were considered to have ratio-only FLC abnormalities and not considered in the evaluation of elevated FLC^13^. We also evaluated the absolute value or sum of the free light chains, which has utility in non-clonal disease situations and has been shown to predict survival in the general population^23^. The timing of the plasma or serum samples was a median of 2 months between diagnosis date and blood draw date for sporadic CLL cases (*N* = 302) (range = (0 years–5.7 years), IQR = (2 weeks–9 months)), and a median 18 months between diagnosis and blood draw date for familial CLL cases (*N* = 154) (range = (0 years–25 years), IQR = (6 months–4.2 years)). Additionally, the FLC was measured in the baseline sample for the majority of subjects, therefore corresponding baseline MBL status was used. Some of the subjects may have had MBL identified in a follow-up sample, but for this study we use the MBL status at the time of the FLC measurement.

### Statistical analyses and considerations

Analyses of FLC levels were conducted between the following groups: (1) Sporadic CLL cases vs. familial CLL cases; (2) Sporadic CLL cases vs. controls; (3) Familial CLL cases vs. controls; (4) Familial MBL vs. controls, and (5) Unaffected relatives in CLL/MBL families vs. controls. Age at FLC was summarized as mean ± standard error (SE), and two-sample *t*-tests were used for age comparisons across groups. Sex and percent elevated FLC were summarized by group as counts and percentages, respectively. The percent of individuals having elevated FLC was compared using age- and sex-adjusted logistic regression models. The FIRTH option^34^ was applied to logistic regression to test for significance when there were small numbers in particular cells and estimates were thought to be unstable. Multivariable linear regression models were used to calculate the least squares (LS) means of *κ*, *λ*, *κ*/*λ* ratios, and FLC sum after adjusting for age and sex; due to the non-normal distributions of the FLC measures, we first log-transformed the values, performed the calculations, then exponentiated the values to summarize.

We performed secondary analyses to ensure that statistical differences were not due to relatedness. We randomly chose one relative from each family from each relative group (either one unaffected or one MBL) and reran analyses above.

Due to the age of the reference population, analyses that compared to the reference group were limited to individuals who were age 50 years and older. Comparisons between sporadic and familial CLL cases included cases who were age 35 and older at sample collection; sensitivity analyses were performed subsetting to age 50 years.

Analyses were performed using SAS version 9.4 (SAS Institute, Cary, NC).

## Results

### Study participants

We identified 302 sporadic CLL cases from MER, 154 familial CLL cases, 87 familial MBL cases, 263 unaffected first-degree relatives from GEC, and 15,396 subjects from the Olmsted County reference population, otherwise referred to as controls.

### Comparison of sporadic CLL and familial CLL cases

Characteristics and comparisons between sporadic CLL and familial CLL cases are shown in Table 1. Sporadic and familial cases were similar in age, however, sporadic cases were comprised of more males (68%) compared to familial cases (49%) (Table 1). The adjusted mean FLC sum (familial = 2.75, sporadic = 2.70; *p*-value = 0.73) and rFLC (familial = 1.03, sporadic = 1.13; *p*-value = 0.42) were similar in both CLL case groups. When investigating the percent elevated FLC (monoclonal and polyclonal combined) by age group, there was no statistically significant difference between familial and sporadic CLL cases. Likewise, when examining specific type of elevation, there were no statistically significant differences between the familial and sporadic cases (*p*’s > 0.05).

**Table 1:** Summary and comparisons of demographic characteristics and serumfree lightchain (sFLC) values in sporadic vs. familial chronic lymphocytic leukemia (CLL) cases, ages 35 and older at sample collection, and Olmsted County controls

	CLL cases		Controls	*p*-values		
	Familial CLL	Sporadic CLL		Familial vs. sporadic CLL cases	Familial CLL vs. controls	Sporadic CLL vs. controls
*N*	154	302	15, 396			
No. of families	118	–				
Age, mean (SE)	64.2 (1.0)	63.2 (0.6)	63.9 (0.1)	0.366	0.003	<0.001
Female, *N* (%)	79 (51.3%)	97 (32.1%)	8,529 (55.4%)	<0.001	0.505	<0.001
sFLC sum, LS mean (SE)	2.75 (1.05)	2.70 (1.04)	2.81 (1.00)	0.730	0.333	0.123
*κ*, LS^a^ mean (SE)	1.22 (1.07)	1.27 (1.05)	1.24 (1.00)	0.638	0.308	0.342
*λ*, LS^a^ mean (SE)	1.19 (1.06)	1.13 (1.04)	1.51 (1.00)	0.493	<0.001	<0.001
rFLC (*κ*:*λ*), LS^a^ mean (SE)	1.03 (1.09)	1.13 (1.07)	0.82 (1.00)	0.420	<0.001	<0.001
Age 35–39 (*N*)	3 (1.9%)	1 (<1%)				
Elevated sFLC	1 (33.3%)	0 (0.0%)		0.982		
Polyclonal sFLC	0 (0.0%)	0 (0.0%)				
Monoclonal sFLC	1 (33.3%)	0 (0.0%)		0.982		
Age 40–49 (*N*)	14 (9.0%)	43 (14.2%)				
Elevated sFLC	4 (28.6%)	9 (20.9%)		0.600		
Polyclonal sFLC	2 (14.3%)	2 (4.7%)		0.201		
Monoclonal sFLC	2 (14.3%)	7 (16.3%)		0.780		
Age 50–59 (*N*)	45 (29.2%)	68 (22.5%)	6,323 (41.1%)			
Elevated sFLC	10 (22.2%)	14 (20.6%)	584 (9.2%)	0.822	0.004	0.006
Polyclonal sFLC	1 (2.2%)	5 (7.4%)	550 (8.7%)	0.392	0.194	0.530
Monoclonal sFLC	9 (20.0%)	9 (13.2%)	34 (0.5%)	0.394	<0.001	<0.001
Age 60–69 (*N*)	43 (27.9%)	112 (37.1%)	4,604 (29.8%)			
Elevated sFLC	12 (27.9%)	31 (27.7%)	747 (16.2%)	0.941	0.060	0.009
Polyclonal sFLC	5 (11.6%)	19 (17.0%)	713 (15.5%)	0.325	0.418	0.968
Monoclonal sFLC	7 (16.3%)	12 (10.7%)	34 (0.7%)	0.225	<0.001	<0.001
Age 70–79 (*N*)	32 (20.8%)	60 (19.9%)	3,121 (20.3%)			
Elevated sFLC	12 (37.5%)	28 (46.7%)	810 (26.0%)	0.671	0.180	0.010
Polyclonal sFLC	6 (18.8%)	13 (21.7%)	775 (24.8%)	0.952	0.369	0.204
Monoclonal sFLC	6 (18.8%)	15 (25.0%)	35 (1.1%)	0.698	<0.001	<0.001
Age 80 + (*N*)	17 (11.0%)	18 (6.0%)	1348 (8.8%)			
Elevated sFLC	10 (58.8%)	8 (44.4%)	567 (42.1%)	0.459	0.182	0.893
Polyclonal sFLC	6 (35.3%)	2 (11.1%)	551 (40.9%)	0.132	0.604	0.012
Monoclonal sFLC	4 (23.5%)	6 (33.3%)	16 (1.2%)	0.463	<0.001	<0.001

Kappa (*κ*) and Lambda (*λ*) are measured mg/L

*CLL* chronic lymphocytic leukemia, *sFLC* serumfree light-chain, *SE* standard error,

*MBL* monoclonal B-cell lymphocytosis, *rFLC* FLC ratio (*κ*:*λ*)

^a^LS-Least squares mean adjusted for age and sex

### Comparison of CLL cases (sporadic/familial) and reference population (controls)

Characteristics and comparisons between CLL cases (familial and sporadic) and controls are shown in Table 1. The mean age of familial CLL cases (64.2 years) was higher than controls (63.9 years), while the mean age of sporadic CLL cases (63.2 years) was slightly lower (*p* < 0.003). There were higher proportions of females in the familial cases (51%) and control groups (55%) compared to sporadic cases (32%) (Table 1). Mean lambda FLC values (λ-FLC; mg/L) were significantly lower in familial (1.19 mg/L) and sporadic (1.13 mg/L) CLL cases compared to the controls (1.51 mg/L) (*p* < 0.001). Mean kappa FLC values (*κ* -FLC; mg/L) were similar across groups: 1.22 mg/L for familial cases, 1.27 mg/L for sporadic cases, and 1.24 mg/L for controls (*p* > 0.05). The rFLC (*κ*:*λ*) was significantly higher in familial (1.03) and sporadic (1.13) CLL cases compared to the controls (0.82) (*p*’s < 0.001). Percent elevated FLC (polyclonal and monoclonal combined) was significantly higher in both familial and sporadic CLL cases compared to the controls in younger age groups (*p* < 0.05). Specifically, in ages 50–59 years, percent elevated FLC was ~12% higher in familial (22%) and sporadic (21%) cases compared to controls (9%) (*p* < 0.01). In ages 60–69 years, percent elevated FLC was ~11% higher in cases compared to controls (16%); this was seen in both sporadic (28%; *p* = 0.009) and familial cases (28%, *p* = 0.06). In ages 70–79 years, percent elevated FLC was significantly higher (~21%) in sporadic cases (47%) compared to controls (26%; *p* = 0.01). Although not statistically significant, familial cases (38%) also had a higher percent elevated FLC (~12%) compared to controls (26%) (*p* = 0.18). However, at older ages (80 + years), we did not see a statistically significant difference between percent elevated FLC in sporadic (44%) or familial (59%) cases compared to controls (42%) (*p* = 0.89 and *p* = 0.18, respectively).

When separating elevated FLC into monoclonal and polyclonal elevation, there was no statistically significant difference between percent of polyclonal FLC in sporadic (7%, 17%, 22%) or familial (2%, 12%, 19%) cases compared to controls (9%, 15%, 25%), for age groups 50–59, 60–69, and 70–79, respectively (*p*’*s* > 0.05). In the oldest age group (80 + years), sporadic CLL cases had lower percent polyclonal FLC (11%) compared to controls (41%; *p* = 0.01), however, the sample size in this group was small (*n* = 2/24). However, a statistically significant (*p* < 0.001) increase in monoclonal FLC elevation was observed in both familial (20%, 16%, 19%, 24%) and sporadic (13%, 11%, 25%, 33%) CLL cases compared to the controls (0.5%, 0.7%, 1%, 1%) across age groups 50–59, 60–69, 70–79, and 80 + , respectively. In a sensitivity analysis of familial and sporadic cases (ages 50 + years at sample collection), results remained similar (Supplementary Table 1).

### Comparisons of familial MBL, unaffected relatives, and reference population (controls)

Characteristics and comparisons between familial MBL, unaffected relatives from CLL/MBL families, and controls are shown in Table 2. Relatives with MBL were on average older (68.4 years) than unaffected family members (63.6 years) or controls (63.9 years); however, the difference was only statistically significant for familial MBL compared to controls (*p* < 0.001). The proportion of females was significantly higher among the unaffected relatives (68%) compared to the controls (55%; *p* < 0.001). Familial MBL cases and unaffected relatives both had a higher age and sex-adjusted mean serum rFLC than the controls, although values were all within the normal range (0.26 to 1.65)^13,33^, (rFLC = 1.04 for familial MBL, 1.00 for unaffected family members, and 0.82 for controls, respectively (*p* < 0.001)). Similar trends were observed when the analyses were repeated using one randomly selected relative from each family per category (Supplementary Table 2).

**Table 2:** Summary and comparisons of demographic characteristics and serumfree lightchain (sFLC) values in 350 relatives, ages 50 and older at sample collection within 155 chronic lymphocytic leukemia/monoclonal B-cell lymphocytosis (CLL/MBL) families vs. 15,396 Olmsted County controls

	Family members		Controls	*p*-values	
	MBL	Unaffected		MBL vs. controls	Unaffected vs. controls
*N*	87	263	15, 396		
Age, mean (SE)	68.4 (1.2)	63.6 (0.7)	63.9 (0.1)	<0.001	0.609
Female, *N* (%)	43 (49.4%)	178 (67.7%)	8,529 (55.4%)	0.264	<0.001
sFLC sum, LS mean (SE)	2.71 (1.05)	2.57 (1.03)	2.81 (1.00)	0.423	<0.001
*κ*, LS^a^ mean (SE)	1.34 (1.06)	1.27 (1.03)	1.24 (1.00)	0.177	0.577
*λ*, LS^a^ mean (SE)	1.29 (1.05)	1.26 (1.03)	1.51 (1.00)	0.001	<0.001
rFLC (*κ*:*λ*), LS^a^ mean (SE)	1.04 (1.04)	1.00 (1.03)	0.82 (1.00)	<0.001	<0.001
Age 50–59 (*N*)	25 (28.7%)	118 (44.9%)	6,323 (41.1%)		
Elevated sFLC	2 (8.0%)	6 (5.1%)	584 (9.2%)	0.999	0.154
Polyclonal sFLC	2 (8.0%)	5 (4.2%)	550 (8.7%)	0.925	0.113
Monoclonal sFLC	0 (0.0%)	1 (0.8%)	34 (0.5%)	0.389	0.275
Age 60–69 (*N*)	21 (24.1%)	76 (28.9%)	4,604 (29.8%)		
Elevated sFLC	3 (14.3%)	11 (14.5%)	747 (16.2%)	0.986	0.951
Polyclonal sFLC	2 (9.5%)	9 (11.8%)	713 (15.5%)	0.614	0.576
Monoclonal sFLC	1 (4.8%)	2 (2.6%)	34 (0.7%)	0.009	0.015
Age 70–79 (*N*)	29 (33.3%)	45 (17.1)	3,121 (20.3%)		
Elevated sFLC	9 (31.0%)	9 (20.0%)	810 (26.0%)	0.898	0.329
Polyclonal sFLC	7 (24.1%)	8 (17.8%)	775 (24.8%)	0.612	0.251
Monoclonal sFLC	2 (6.9%)	1 (2.2%)	35 (1.1%)	0.012	0.250
Age 80 + (*N*)	12 (13.8%)	24 (9.1%)	1,348 (8.8%)		
Elevated sFLC	6 (50.0%)	12 (50.0%)	567 (42.1%)	0.774	0.559
Polyclonal sFLC	5 (41.7%)	10 (41.7%)	551 (40.9%)	0.845	0.890
Monoclonal sFLC	1 (8.3%)	2 (8.3%)	16 (1.2%)	0.008	0.002

Kappa (*κ*) and Lambda (*λ*) are measured mg/L

*CLL* chronic lymphocytic leukemia, *sFLC* serumfree lightchain, *SE* standard error, *MBL* monoclonal B-cell lymphocytosis, *rFLC* FLC ratio (*κ*:*λ*)

^a^LS-least squares mean adjusted for age and sex

When investigating the percent elevated FLC by age group (50–59, 60–69, 70–79, and 80+), there were no statistically significant differences between familial MBL (8%, 14%, 31%, 50%) or unaffected relatives (5%, 14%, 20%, 50%) compared to controls (9%, 16%, 26%, 42%). However, when looking at types of FLC elevation, there were statistically significant differences in monoclonal, but not polyclonal, elevation in family members in most of the age groups 60 and older, including MBL cases (5%, 7%, 8%) and unaffected relatives in CLL / MBL families (3%, 2%, 8%), compared to the Olmsted County controls (2%, 1%, 1%) for ages 60–69, 70–79 and 80+, respectively (all *p*’s < 0.05); the only exception was the age 70–79 group of unaffected relatives compared to controls (*p* = 0.25). Analyses restricted to only one family member showed generally similar results, although statistical significance varied by age group (Supplementary Table 2).

## Discussion

We demonstrate increased monoclonal elevations for CLL cases, regardless of family history, compared to a reference population. Monoclonal elevation in both sporadic and familial CLL cases was higher than in the reference population. We also found evidence for increased monoclonal elevation in relatives, including unaffected family members as well as those with MBL, compared to that of the reference population, which may provide additional risk information in unaffected and MBL relatives of CLL probands.

We report no differences in sFLC elevation between familial and sporadic CLL cases, which is consistent with prior reports that have demonstrated little or no difference in other clinical and prognostic markers between these two groups^19,20,35–38^. For example, no differences in the expression of biologic markers (ZAP-70, CD38, and CD23), levels of serum β2-microglobulin, *CXCR4* expression, or chromosome 13q deletion have been reported^20,37,38^. Thus, our results from this study add to evidence for similar biology underlying sporadic and familial CLL.

Our finding of an increased monoclonal, but not polyclonal, protein elevation among unaffected family members compared to the controls is provocative. Polyclonal FLC elevation, where the rFLC is normal, can be due to a number of causes including renal dysfunction, inflammation, or immune stimulation^39,40^, whereas a monoclonal elevation is suggestive of clonal plasma cell proliferation (e.g., multiple myeloma/MGUS)^41,42^. In a prior study of CLL, the monoclonal elevated light chain in the serum (kappa or lambda) agreed with the clonal B-cell’s lightchain restriction by flow cytometry in 96% of cases^13,18^. In addition to elevated sFLC measures in our unaffected relatives, we also observed elevated sFLC measures in the familial MBL cases compared to a population-based reference. It has been shown in a prospective study that an abnormal rFLC can be present several years before the actual diagnosis of CLL in a significant percentage of patients^18^. Taken together, these findings suggest that monoclonal sFLC elevation may identify a precursor clonal process prior to the onset of CLL. Confirmation in other studies is necessary, but results could suggest incorporating FLC measures and MBL status (enhanced testing for MBL clones) to inform family members at greatest risk of progression to CLL, especially among those who are 60 years and older.

Our study has a number of strengths, including a well-characterized collection of familial and sporadic CLL cases, all of whom had validated CLL diagnoses through medical record review, and a large comparable and systematically screened population-based reference group. Limitations include the small sample size for some of the age groups examined and generalizability to other ethnicities due to the primarily Caucasian nature of our families and Olmsted County reference population (controls). Additionally, we make the assumption that the Olmsted County population is unaffected, as they are not screened for MBL, however to the best of our knowledge they have no known reported CLL. Regarding the unaffected relatives from the GEC study sites, primarily the same baseline sample was used for the sFLC measurements and the MBL screening, however, there were some samples that did not get screened for MBL and were assumed to be unaffected. Finally, we did not have total serum protein or the flow cytometry results available to correlate the lightchain restriction pattern with the sFLC results. However, prior literature suggests high concordance between monoclonal elevated light chain in serum and the CLL clone’s lightchain restriction^13,18^. Another important consideration is we were unable to perform analyses including other prognostic parameters due to missingness for familial CLL cases. Future studies should aim to consider prognostic parameters (e.g., beta-2-microglobulin, Rai Stage, IgHV mutational status, FISH cytogenetics, CLL-IPI index) with sFLC in sporadic vs. familial context.

Our study showed that sporadic and familial CLL have similar sFLC but increased monoclonal sFLC elevation compared to controls. In addition, monoclonal elevation of sFLC was higher among unaffected relatives and relatives with MBL from CLL families than a general population. To our knowledge, this is one of the first studies to examine sFLC among relatives from CLL/MBL families and provide further evidence of the potential of monoclonal sFLC elevations as a valuable prognostic factor in CLL/MBL. Follow-up studies are needed to replicate our findings and determine the relationship between elevated sFLC levels, MBL, and future CLL risk.

## Supplementary information

Supplemental Material
