# Supplementary material for: Association of elevated serumfree light chains with chronic lymphocytic leukemia and monoclonal B-cell lymphocytosis
Source: Blood Cancer J. 2019 Aug 5;9(8):59. doi: 10.1038/s41408-019-0220-x (PMC6683199; doi:10.1038/s41408-019-0220-x)
Supplement: Supplementary file 1 — Supplemental Material [file 41408_2019_220_MOESM1_ESM.docx]

| **Supplementary Table 1**. Summary and comparisons of demographic characteristics and serum free light chain (sFLC) values in 137 CLL cases within 106 Chronic Lymphocytic leukemia / Monoclonal B-cell lymphocytosis (CLL / MBL) families and 258 sporadic CLL cases versus 15,396 Olmsted County Controls. Subjects are all age 50+ years at sample collection. | | | | | |
| --- | --- | --- | --- | --- | --- |
|  | **CLL Cases** | | **Controls** | **P-values** | |
|  | **Familial CLL** | **Sporadic CLL** |  | **Familial CLL vs Controls** | **Sporadic CLL vs Controls** |
| **N** | 137 | 258 | 15,396 |  |  |
| ***Age, Mean (SE)*** | 66.5 (0.9) | 66.1 (0.5) | 63.9 (0.1) | 0.003 | <0.001 |
| ***Female, N (%)*** | 72 (52.6%) | 81 (31.4%) | 8529 (55.4%) | 0.505 | <0.001 |
| ***sFLC Sum, LS Mean (SE)*** | 2.71 (1.04) | 2.69 (1.03) | 2.81 (1.00) | 0.333 | 0.123 |
| **κ, LS* Mean (SE)** | 1.19 (1.04) | 1.28 (1.03) | 1.24 (1.00) | 0.308 | 0.342 |
| **λ, LS* Mean (SE)** | 1.20 (1.04) | 1.13 (1.03) | 1.51 (1.00) | <0.001 | <0.001 |
| **rFLC (κ:λ), LS* Mean (SE)** | 0.99 (1.04) | 1.13 (1.03) | 0.82 (1.00) | <0.001 | <0.001 |
| **Age 50-59 (N)** | 45 (32.8%) | 68 (26.4%) | 6323 (41.1%) |  |  |
| Elevated sFLC | 10 (22.2%) | 14 (20.6%) | 584 (9.2%) | 0.004 | 0.006 |
| Polyclonal sFLC | 1 (2.2%) | 5 (7.4%) | 550 (8.7%) | 0.194 | 0.530 |
| Monoclonal sFLC | 9 (20.0%) | 9 (13.2%) | 34 (0.5%) | <0.001 | <0.001 |
| **Age 60-69 (N)** | 43 (31.4%) | 112 (43.4%) | 4604 (29.8%) |  |  |
| Elevated sFLC | 12 (27.9%) | 31 (27.7%) | 747 (16.2%) | 0.060 | 0.009 |
| Polyclonal sFLC | 5 (11.6%) | 19 (17.0%) | 713 (15.5%) | 0.418 | 0.968 |
| Monoclonal sFLC | 7 (16.3%) | 12 (10.7%) | 34 (0.7%) | <0.001 | <0.001 |
| **Age 70-79 (N)** | 32 (23.4%) | 60 (23.3%) | 3121 (20.3%) |  |  |
| Elevated sFLC | 12 (37.5%) | 28 (46.7%) | 810 (26.0%) | 0.180 | 0.010 |
| Polyclonal sFLC | 6 (18.8%) | 13 (21.7%) | 775 (24.8%) | 0.369 | 0.204 |
| Monoclonal sFLC | 6 (18.8%) | 15 (25.0%) | 35 (1.1%) | <0.001 | <0.001 |
| **Age 80+ (N)** | 17 (12.4%) | 18 (7.0%) | 1348 (8.8%) |  |  |
| Elevated sFLC | 10 (58.8%) | 8 (44.4%) | 567 (42.1%) | 0.182 | 0.893 |
| Polyclonal sFLC | 6 (35.3%) | 2 (11.1%) | 551 (40.9%) | 0.604 | 0.012 |
| Monoclonal sFLC | 4 (23.5%) | 6 (33.3%) | 16 (1.2%) | <0.001 | <0.001 |
| CLL=Chronic Lymphocytic Leukemia, sFLC= serum free light chain, SE=Standard Error,  MBL= Monoclonal B-cell lymphocytosis  *LS-Least squares mean adjusted for age and sex  Kappa (κ) and Lambda (λ) are measured mg/L  rFLC=FLC ratio (κ:λ) | | | | | |

| **Supplementary Table 2.** Summary and comparisons of demographic characteristics and serum free light chain (sFLC) values in one randomly selected subject per group per Chronic Lymphocytic leukemia / Monoclonal B-cell lymphocytosis (CLL/MBL) family versus Olmsted County Controls | | | | | |
| --- | --- | --- | --- | --- | --- |
| **CLL /MBL**  **(N= 155 families)** | **Family members** | | **Controls** | **P-values** | |
|  | **MBL** | **Unaffected** |  | **MBL vs controls** | **Unaffected vs controls** |
| **N** | 59 | 139 | 15,396 |  |  |
| ***Age, Mean (SE)*** | 69.8 (1.5) | 63.2 (0.9) | 63.9 (0.1) | <0.001 | 0.382 |
| ***Female, N (%)*** | 31 (52.5%) | 95 (68.3%) | 8529 (55.4%) | 0.660 | 0.002 |
| ***sFLC Sum, LS Mean (SE)*** | 2.71 (1.06) | 2.68 (1.04) | 2.81 (1.00) | 0.530 | 0.201 |
| **κ, LS* Mean (SE)** | 1.36 (1.07) | 1.31 (1.04) | 1.24 (1.00) | 0.190 | 0.219 |
| **λ, LS* Mean (SE)** | 1.28 (1.06) | 1.32 (1.04) | 1.51 (1.00) | 0.005 | <0.001 |
| **rFLC (κ:λ), LS* Mean (SE)** | 1.06 (1.05) | 0.99 (1.03) | 0.82 (1.00) | <0.001 | <0.001 |
| **Age 50-59 (N)** | 15 (25.4%) | 59 (42.4) | 6323 (41.1%) |  |  |
| Elevated sFLC | 1 (6.7%) | 3 (5.1%) | 584 (9.2%) | 0.968 | 0.308 |
| Polyclonal sFLC | 1 (6.7%) | 3 (5.1%) | 550 (8.7%) | 0.974 | 0.363 |
| Monoclonal sFLC | 0 (0.0%) | 0 (0.0%) | 34 (0.5%) | 0.241 | 0.741 |
| **Age 60-69 (N)** | 14 (23.7%) | 47 (33.8%) | 4604 (29.8%) |  |  |
| Elevated sFLC | 2 (14.3%) | 5 (10.6%) | 747 (16.2%) | 0.978 | 0.464 |
| Polyclonal sFLC | 2 (14.3%) | 3 (6.4%) | 713 (15.5%) | 0.918 | 0.153 |
| Monoclonal sFLC | 0 (0.0%) | 2 (4.3%) | 34 (0.7%) | 0.299 | 0.002 |
| **Age 70-79 (N)** | 20 (33.9%) | 22 (15.8%) | 3121 (20.3%) |  |  |
| Elevated sFLC | 6 (30.0%) | 5 (22.7%) | 810 (26.0%) | 0.942 | 0.722 |
| Polyclonal sFLC | 5 (25.0%) | 4 (18.2%) | 775 (24.8%) | 0.867 | 0.467 |
| Monoclonal sFLC | 1 (5.0%) | 1 (4.5%) | 35 (1.1%) | 0.068 | 0.041 |
| **Age 80+ (N)** | 10 (16.9%) | 11 (7.9%) | 1348 (8.8%) |  |  |
| Elevated sFLC | 5 (50.0%) | 5 (45.5%) | 567 (42.1%) | 0.836 | 0.786 |
| Polyclonal sFLC | 4 (40.0%) | 4 (36.4%) | 551 (40.9%) | 0.740 | 0.831 |
| Monoclonal sFLC | 1 (10.0%) | 1 (9.1%) | 16 (1.2%) | 0.005 | 0.009 |
| CLL=Chronic Lymphocytic leukemia, sFLC= serum free light chain, SE=Standard Error, MBL=Monoclonal B-cell lymphocytosis  *LS-Least squares mean adjusted for age and sex  Kappa (κ) and Lambda (λ) are measured mg/L  rFLC=FLC ratio (κ:λ) | | | | | |
